# Understanding the needs for support and coping strategies in grief following the loss of a significant other: insights from a cross-sectional survey in Sweden

**DOI:** 10.1177/26323524241275699

**Published:** 2024-09-08

**Authors:** Inger Benkel, Johanna Skoglund, Daniel Enstedt, Ylva Hård af Segerstad, Joakim Öhlén, Stina Nyblom

**Affiliations:** Palliative Centre, Högsbo Hospital, Box 30110, Gothenburg 40043, Sweden; Institute of Medicine, Sahlgrenska Academy, University of Gothenburg, Gothenburg, Sweden; Palliative Centre at Sahlgrenska University Hospital Region Västra Götaland, Gothenburg, Sweden; Palliative Centre at Sahlgrenska University Hospital Region Västra Götaland, Gothenburg, Sweden; Religious Studies, Department of Literature, History of Ideas and Religion, University of Gothenburg, Gothenburg, Sweden; Department of Applied IT and Centre for Person-Centred Care, University of Gothenburg, Gothenburg, Sweden; Palliative Centre at Sahlgrenska University Hospital Region Västra Götaland, Gothenburg, Sweden; Institute of Health and Care Sciences and Centre for Person-Centred Care, University of Gothenburg, Gothenburg, Sweden; Institute of Medicine, Sahlgrenska Academy, University of Gothenburg, Gothenburg, Sweden; Palliative Centre at Sahlgrenska University Hospital Region Västra Götaland, Gothenburg, Sweden

**Keywords:** bereavement, coping, family, grief, support in bereavement

## Abstract

**Background:**

Grief has previously been described in pathological terms, characterized by several stages. In the past three decades, new perspectives on grief as a reaction to the loss of a significant other have emerged. It shows that grief is an individual process based on circumstances surrounding the death and the bereaved person’s life situation, rather than being predetermined.

**Objective:**

The aim of the study was to show how grief is perceived by people who have lost a significant other, and it focuses on bereavement support, how the death affects the bereaved person’s living conditions, how the bereaved person deals with grief, and if grief is expressed differently depending on whether it was an expected death (ED) or an unexpected death (UED).

**Design:**

A cross-sectional design was used with data collected anonymously using an online survey with semi-structured answers and options for participants to add their own comments, and it was analyzed descriptively.

**Result:**

Support in grief was mainly given by family and friends, and the perceived need was primarily for emotional support or emotional support combined with practical support, and to a greater extent for UEDs and women. For some bereaved persons, health caregivers and religious institutions provided support outside their own network. Grief can affect how people socialize with others and change social relationships. People can deal with grief in social as well as religious ways in the company of friends, through everyday conversations, spending time in nature, and having a spiritual outlook on life, and with the help of pets.

**Conclusion:**

The results can contribute to an increased understanding of grief after the loss of a significant other and how grief affects the bereaved person’s life depending on whether it is an ED or a UED. There was a difference between the genders, with women perceiving a need for and receiving different forms of support and to a greater extent than men.

## Plain language summary

Understanding support and coping in grief

Grief has long been described as a mental health disorder and characterized by multiple stages. In the last three decades, research has shown new perspectives on grief as a normal reaction to the loss of a significant other. It has shown that grief is an individual process based on the circumstances surrounding the death, the bereaved person’s social life situation, different forms of support and how the bereaved person deals with their changed life situation. The aim of this study was to investigate what support in grief can look like and the coping strategies that are used. The grieving process can vary depending on how the death affects the bereaved person’s living conditions, how they deal with grief, and whether it was perceived as an expected death (ED) or an unexpected death (UED). The study took the form of an online survey with data collected as anonymous semi-structured answers, with options for participants to add their own comments, and it was analysed descriptively. The results showed that support in grief was mainly provided by family and friends, regardless of whether it was an ED or a UED. For some bereaved persons, health caregivers and religious institutions provided support outside their own network. The participants mainly reported a need for emotional support and emotional support combined with practical support. Grief can influence how people socialize with others and lead to changes in social relationships. People can cope with grief in social and religious ways, by socializing with friends, and through everyday conversations, spending time in nature and having a spiritual outlook on life, and with the help of pets. The conclusions drawn from the results can contribute to a greater understanding of the bereavement of a significant other and how grief influences the life circumstances of a bereaved person in many ways.

## Introduction

Historical notions of grief in pathological terms or as a mental disorder characterized by specific stages are still common, in particular in popularized discourses. In this old view, the final stage of the process, a “closure” of the relationship with the sick person should be achieved, ending the grieving process. This perspective has perpetuated the notion that the grieving process is the same for everyone, “one size fits all.”^1–4^ However, this view of grief is largely based on myth, poorly supported by evidence, and has been challenged by contemporary research.^5,6^ Over the last three decades, new perspectives on grief, as a reaction to the loss of a significant other, have emerged suggesting that grief is an individual and natural process based on the circumstances surrounding the death and the bereaved person’s life situation, rather than being predetermined.^7–10^ In the new view of grief, it is affected by all aspects of life, including physical, psychological, social, cognitive, and existential factors, as well as the cause of death and whether it was expected or not.^11–15^

It is argued that bereavement should be seen in terms of resilience to changes in life circumstances, rather than likened to recovery from a mental disorder.^16–18^ An individual’s capacity for resilience is based on their previous life experiences and intrapsychic development. Those experiencing bereavement can gain a different outlook on life based on their changed circumstances and new experiences.^16,19^ Some similarities can be found between how a bereaved person deals with grief and how they cope with other upheavals in life and their strategies for doing so.^20–22^

Access to social support and a focus on the present is suggested as being key to an individual’s ability to handle difficult life events, such as a terminal disease or the loss of a significant other.^20,23^ The dual process is described as a strategy for coping with grief in which the grieving person alternates between a loss-oriented approach, when they focus on the loss, its consequences, and the emotions that accompany it, and a restorative approach, when their focus is beyond the loss, on what is to come.^24^ If the bereaved person is part of a family, the family as a whole grieves. “Family grief” affects the individual and vice versa.^22,25^ Religious coping distinguishes between *collaborative*, *self-directing*, and *deferring* coping styles. Collaborative religious coping involves an active partnership with the divine, seeking guidance and support. Deferring religious coping, on the other hand, involves relying on a higher power to manage the situation without active personal involvement, while the self-directing style includes active coping orientation, which emphasizes personal agency, rather than traditional religious connection.^26,27^ Importantly, Pargament’s model can be integrated with other coping strategies, both religious and non-religious.

Support for people experiencing bereavement, both practical and emotional, is usually provided by close family and friends and is often sufficient for the bereaved, and professional support is therefore not needed.^28,29^ In this context, family members include direct and indirect relatives as well as friends with a strong relationship or connection to the deceased. This can present some difficulties when it comes to defining the network and who is available to provide support.^28–30^ The Irish Hospice Foundation has developed a bereavement pyramid that describes that few persons need support from professionals in their bereavement process.^31^ Furthermore, the Internet and various social media platforms offer new possibilities for bereaved individuals to engage in online grief support communities, in which they can find social support from peers who share similar experiences of loss and grief.^32–34^

Despite the new perspective on grief, many professionals argue that grief can be considered a mental disorder, and the past perspectives on bereavement continue to influence the treatment and understanding of grief even today.^35,36^ This study is part of a broader project that aims to contribute empirical knowledge about how grief is expressed by people in Sweden who have lost a significant other. The article focuses on bereavement support and how death affects the living conditions of the bereaved person, how they deal with grief, and whether grief expresses itself differently depending on whether the death was expected (ED) or unexpected (UED).

## Method

### Design

The study uses a cross-sectional design with data collected anonymously from a web-based, semi-structured, mixed questionnaire, which also included the option for participants to add their own comments to the questions.^37^ In total, the survey comprised 54 questions related to grief following the death of a significant other. This article is part of a broader project that includes reporting on 20 questions about support and coping in bereavement and how death impacts the life circumstances of people experiencing bereavement. It also includes questions about helpful factors when dealing with grief as well as measuring religious and spiritual practice and attendance. Through the survey, participants could provide reflections on sources of support when experiencing grief following the death of someone close to them.

### Participants and recruitment

Participants in the study were recruited in collaboration with two civil society associations in Sweden, a bereavement support association and a pensioners’ association in West Sweden. To be included, participants had to be over 18 and have experienced the death of a significant other. They also had to be able to complete a questionnaire in Swedish.

### Data collection

An invitation to participate in the study was sent via a digital newsletter to members of the associations. The invitation included information about the study, contact information for the research team, and a link to the web-based questionnaire. The questionnaire was open to participants between February and May 2022, and all responses were collected during this period. No follow-up was possible due to the survey being anonymous. The exact number of people who received the invitation is not clear, which makes it difficult to estimate the response rate for the questionnaire. No reminder about the survey was sent out.

### Data analysis

The data generated from the questionnaire were stored on secure storage provided by Sahlgrenska University Hospital and analyzed using descriptive statistics comparing similarities and differences between outcomes based on ED versus UED. A descriptive analysis was conducted of the open-ended responses against the question topics. The most salient aspects of grief were described and compared against each other, and supporting quotes were linked.^38,39^ The reporting of this study conforms to the STROBE statement checklist (Supplemental Material).

### Ethics

On the understanding that the questions could provoke reflection on difficult feelings and thoughts, the announcement stated that the research team could, if necessary, help the participants with reference to appropriate support. It also stated that participation in the study could be ended at any time, in which case the participant’s data would be automatically deleted. The participants could also choose to skip questions in the survey, for example, if they found them painful to answer or if they did not apply to them.

## Results

A total of 255 participants (181 women and 74 men) answered the survey, all of whom had lost a significant other 6 months to 5 years prior. None of the participants had experienced a loss less than 6 months prior. The age of the participants ranged from 22 to 91 and the majority were older than 65, meaning they were most likely retired (<65 years = 81; >65 = 170). For more information, see Table 1.

**Table 1.** Characteristics of the study participants.

Variable	Expected death (*n* = 134)	Unexpected death (*n* = 121)
Participant background		
Gender		
Men	47/134 (35.1%)	27/121 (22.3%)
Women	87/134 (64.9%)	94/121 (77.7%)
Geographical area of residence		
City	19/134 (14.2%)	29/121 (24.0%)
Large urban area	64/134 (47.8%)	36/121 (29.8%)
Small urban area/rural area	51/134 (38.1%)	56/121 (46.3%)
Education		
Primary school	20/134 (14.9%)	12/121 (9.9%)
Upper secondary school	42/134 (31.3%)	35/121 (28.9%)
University	72/134 (53.7%)	74/121 (61.2%)
Employment		
Employed	23/132 (17.4%)	58/121 (47.9%)
Sick leave	1/132 (0.8%)	5/121 (4.1%)
Retired	109/132 (82.6%)	61/121 (50.4%)
Student	2/133 (1.5%)	2/121 (1.7%)
Other	1/132 (0.8%)	2/121 (1.7%)
Living situation		
Living alone	72/125 (57.6%)	52/108 (48.1%)
Cohabiting	53/125 (42.4%)	56/108 (51.9%)
Missing	9 (7%)	13 (10%)
Relationship to the deceased		
Married/cohabiting/partner	71/133 (53.4%)	38/121 (31.4%)
Living apart	1/133 (0.8%)	3/121 (2.5%)
Child/stepchild	22/133 (16.5%)	29/121 (24.0%)
Parent/step-parent	19/133 (14.3%)	33/121 (27.3%)
Sibling	9/133 (6.8%)	12/121 (9.9%)
Relative	2/133 (1.5%)	5/121 (4.1%)
Friend	6/133 (4.5%)	0/121 (0.0%)
Neighbor	3/133 (2.3%)	0/121 (0.0%)
Other	0/133 (0.0%)	1/121 (0.8%)
Missing	1 (0.1%)	0 (0%)

The responses cover topics such as who provided support, need for support, impact of grief on life, social relationships, and help in dealing with grief.

### Who provided support in grief

Suggestions for various forms of support were provided by personal networks, healthcare providers, and religious institutions. The majority of the participants felt that they received the most support from their family and friends, regardless of whether the death was expected or unexpected. More women than men felt that they received support. One woman, aged 68, stated that she had “*received good support from [her] immediate family, children, siblings, and sister and brother-in-law*.”

Participants who had experienced a UED of someone close were more likely to feel that they received support (UED 65% family, 48% friends) than those who experienced an ED (ED 30% family, 23% friends). Those who experienced a UED also received support to a greater extent from people outside their family and friends. Participants emphasized the significance of connecting with other people who had experienced similar instances of grief, expressing the importance of hearing about their resilience and first-hand accounts of their experiences (UED 48%, ED 15%). As one woman, aged 64, expressed: “*Getting to meet other mothers who survived their grief was important to me. Seeing them with my own eyes and hearing them tell me about what they had been through was incredible*.” Those who had experienced an ED reported support from health social workers and registered nurses more frequently than those who had experienced a UED. For more information, see Table 2.

**Table 2.** Who do you feel has provided support in grief? (multiple answers possible).

Variable	Gender			
	Men		Women	
	Expected death	Unexpected death	Expected death	Unexpected death
	*n* = 47	*n* = 27	*n* = 87	*n* = 94
Close family/significant others	5/44 (11.4%)	13/27 (48.1%)	33/85 (38.8%)	64/92 (69.6%)
Friends	4/44 (9.1%)	7/27 (25.9%)	26/85 (30.6%)	50/92 (54.3%)
Co-workers	0/44 (0.0%)	3/27 (11.1%)	9/85 (10.6%)	29/92 (31.5%)
Manager	0/44 (0.0%)	2/27 (7.4%)	4/85 (4.7%)	20/92 (21.7%)
Occupational health services	0/44 (0.0%)	1/27 (3.7%)	1/85 (1.2%)	5/92 (5.4%)
Support group	3/44 (6.8%)	8/27 (29.6%)	18/85 (21.2%)	39/92 (42.4%)
Others who share similar experiences	3/44 (6.8%)	9/27 (33.3%)	17/85 (20.0%)	48/92 (52.2%)
Health social worker	5/44 (11.4%)	2/27 (7.4%)	14/85 (16.5%)	10/92 (10.9%)
Psychologist	2/44 (4.5%)	4/27 (14.8%)	7/85 (8.2%)	21/92 (22.8%)
Psychotherapist	1/44 (2.3%)	1/27 (3.7%)	6/85 (7.1%)	12/92 (13.0%)
Registered nurse	2/44 (4.5%)	0/27 (0.0%)	2/85 (2.4%)	1/92 (1.1%)
Physician	0/44 (0.0%)	0/27 (0.0%)	1/85 (1.2%)	10/92 (10.9%)
Religious representative	2/44 (4.5%)	3/27 (11.1%)	4/85 (4.7%)	19/92 (20.7%)
Judicial representative	0/44 (0.0%)	0/27 (0.0%)	1/85 (1.2%)	2/92 (2.2%)
Other	1/44 (2.3%)	0/27 (0.0%)	2/85 (2.4%)	2/92 (2.2%)
Missing	3 (6%)	1 (3%)	3 (3%)	4 (4%)

### Perceived support needs in grief

Participants who had experienced a UED had a greater need for support (83%) than those who had experienced an ED (44%). Men *did not* expect support (UED 33%, ED 74%) to the same extent as women (UED 12%, ED 46%). Whether they had experienced an ED or a UED, the majority of participants, both men and women, felt that they received the support they needed (ED 71% and UED 61%).

The participants described a variety of different forms of support that were offered. They mainly reported needing emotional support (UED 38%, ED 25%) and emotional support combined with practical support (UED 43%, ED 20%). Emotional support in the form of conversation included individual conversations, either within their network or with a professional, or participation in some form of support group for grief counseling. “*My way of processing grief was by talking to family and friends as well as colleagues*,” woman, aged 60. Those who had experienced a UED were twice as likely (64%) to report a need for emotional support combined with practical support, including advice and information, compared to those who had experienced an ED (31%). The difference in the reported need for only emotional support was smaller between the groups, UED 36% and ED 25%, respectively.

In both groups, about 30% felt that they did not receive the support they needed and expected. This included, among other examples, practical support in daily life that did not materialize and people who did not call as expected. Some expressed expectations that the healthcare system would provide outreach activities, including information or referrals to other support organizations, or follow-up services for those experiencing grief: “*I would have needed more practical support help with childcare. . . even practical things in the home like cleaning. . . I often just didn’t have the energy*,” woman, aged 49. “*Above all, I would have liked to have received more help from society with everything that needs to be taken care of when a significant other passes away, i.e. practical support with what needs to be taken care of, but also for counselling*,” woman, aged 27.

### Impact of grief on life circumstances

With respect to the impact of grief on health, 71% of the participants who experienced an ED described their health as unchanged. Among those who experienced a UED only 38% stated that their health was unchanged. For the participants who had experienced a UED, 61% stated that their health had become worse or much worse, while only 22% of the participants who had experienced an ED described deteriorating health.

The impact of the change in life circumstances was evident for several participants on both the practical aspects of their everyday lives and their emotional well-being. Individuals who had previously shared their lives with the deceased felt a profound sense of loneliness, grappling with the challenge of forging a new, solitary path and envisioning an uncertain future. “*Having lived with someone for a long time and suddenly being alone is extremely difficult. It takes a long time to get used to it. I feel lost and don’t really know what I want to do with the rest of my life*,” woman, aged 57. Others described a contrasting experience, discovering new-found independence and seeking new experiences through travel, despite their loss. “*I am more independent and have travelled more in the world than I think we would have if my husband was still alive*,” woman, aged 60. Notably, among participants who experienced a UED, 57% needed assistance in managing daily chores and maintaining their homes as a result of their grief; only 38% of those who experienced an ED reported the same. It was observed that women exhibited a greater need for practical support in their daily lives compared to men (women UED 68%, ED 28%; men UED 35%, ED 12%).

Most participants reported only a minor impact on their financial (ED 65%, UED 57%) and housing situation (ED 94%, UED 97%) after their loss. However, here gender differences emerged, with women experiencing greater negative effects on their financial situation than men (ED men 11%, women 25%; UED men 14%, women 35%).

For most of the participants, religious activity and spiritual practices played a minor role. Approximately half reported belonging to a religion. A further 18% identified as atheists and 21% as agnostics. A slightly lower proportion reported belonging to a religion or spiritual group (51%) than embracing some form of religious or spiritual belief (64%).

Some participants mentioned receiving religious support, primarily from support groups run by the Church of Sweden, priests, and deacons, although a few stated that these had not been helpful. Some distanced themselves from religious representatives: “*I did not want to talk to any priest or deacon*,” a woman, aged 84. Others ascribed great significance to their religious faith: “*I have lost faith in people. This experience has shown me how bad most of us are at taking care of each other. Without my belief in Jesus Christ, I would probably have been a mental wreck by now, deep in the darkest abyss*,” man, aged 41.

### The impact of grief on social relationships

In some cases, grief impacted participants’ social relationships. For those participants who experienced an ED, 40% felt that grief had affected their social relationships with others greatly or in some way. For the participants who experienced a UED, 76% felt that their relationships were affected greatly or in some way. Most of the participants who experienced an ED (60%) did not experience any change in their social relationships. In contrast, the same was true for only a minority of participants who had experienced a UED (24%).

### Socializing with others

Grief can influence how people socialize with others. Most of the participants spent more time with family and friends after their loss, regardless of the nature of the death (ED 91%, UED 98%). Almost half of the participants, regardless of the nature of the death (ED 51%, UED 42%), stated that they socialized more with new acquaintances than before. More than half of the participants experienced that grief made it difficult to socialize with certain people, although this differed significantly between the two groups (ED 90%, UED 43%), and nearly twice as many in the group who had experienced an ED said that this was the case. This change may be due to the difficulty of meeting people with whom they used to socialize or that other people found it difficult to meet the bereaved person in the absence of the deceased in various contexts: “*Friends with whom I used to spend a lot of time, and who are still together, I now rarely meet and no longer offer invitations. Now I spend most of my time with friends, old and new, and I’m happy with that*.” Woman, aged 80.

About half of the participants in both groups (ED 47%, UED 51%) perceived that reactions from other people were mostly supportive. Some participants felt that there was no change at all in the reactions of other people, and this was nearly double for those who had experienced an ED (36%) compared to a UED (19%). Men, in particular, stated that the reactions of others were unchanged (men ED 45%, UED 35%; women ED 30%, UED 14%). Many participants stated that they felt some people avoided them at some level (ED 61%, UED 74%). Very few participants stated that they experienced intrusiveness from others (ED 13%, UED 21%).

### Help to deal with grief

Engaging with friends, everyday conversations, and spending time in nature stand out when it comes to strategies for coping with grief. As one woman, aged 76, said: “*Being in nature completely without demands, taking a peaceful stroll, was very important to me in the first years after the death, and still is*.” In addition to conversations and social contact, the presence of pets was mentioned: “*I have a dog, which is a great support*,” Woman, aged 69. Sometimes this support is coupled with time spent in nature: “*My dog has been incredibly important, both for comfort and during long walks in the forest*,” Woman, aged 73. Furthermore, one respondent noted emphatically: “*The greatest help in grief was my cat. I believe many pet owners feel the same way as I do*,” Woman, aged 70. “*My cat who is close and wants/needs company and lots of love*,” Woman, aged 68.

Many of the participants (38%–47%) valued various types of activities as a source of support. Examples included physical activity, being part of an association, or personal work. Other activities included engaging in art, literature, and music, and with support groups on the Internet (11%–34%). Many described certain activities as providing a rest from the grief, allowing them to engage in something that offered detachment. Work was considered to be more helpful by those who experienced a UED (48%) than by those who experienced an ED (30%). One woman, aged 57, expressed this as follows: “*Work is the only place where everything is as normal. There I have been able to ‘pretend’ that everything is normal*.” Art, literature, and music were seen as a source of support by some of the participants (men UED 31%, ED 4%; women UED 21%, ED 28%). Online support groups were more helpful for women (men UED 12%, ED 2%; women UED 38%, ED 15%). Other aspects of life that were referenced as being helpful in grief could be meditation, relaxation exercises, hobbies, religious activities, social media, and pets: “*For me, music is a source of relief, as is trying to stay active in associations and going on outings, to the cinema to the theatre*,” Woman, aged 70.

A religious coping style was used by 9% of the participants who put their trust in God and/or a higher power (deferring coping style). A majority (79%) relied on their own ability (self-directing coping style). The remainder (12%) relied on a collaborative coping style in which they put their trust in themselves and God and/or a higher power to assist in times of hardship and crisis. Of the participants who put their trust in their own ability (self-directing coping style), 41% belonged to a religious group. Of those, 39% were Christian, and the remaining participants belonged to other faiths, atheism, or agnosticism. Among those who indicated a self-directing coping style, 45% expressed a belief in God or a higher power, 39% believed in a higher power, 5% believed in God, and 10% believed in both God and a higher power.

Religious activity was deemed important in the coping process for 9% of the participants, while “spiritual practices, such as meditation,” were important for 14% and “relaxation exercises” for 8%. Breaking down the responses based on gender and for ED and UED, women who experienced UED stand out. Among this group, 27% referenced “spiritual practices, such as meditation,” and 17% “relaxation exercises” as assisting them in their grief. See Table 3.

**Table 3.** What has helped you in grief? (multiple answer options possible).

Variable	Gender			
	Men		Women	
	Expected death	Unexpected death	Expected death	Unexpected death
	*n* = 47	*n* = 27	*n* = 87	*n* = 94
Coping in bereavement				
Work	11/44 (25.0%)	12/26 (46.2%)	28/85 (32.9%)	44/92 (47.8%)
Spiritual practices such as meditation	2/44 (4.5%)	3/26 (11.5%)	9/85 (10.6%)	25/92 (27.2%)
Relaxation exercises	1/44 (2.3%)	2/26 (7.7%)	5/85 (5.9%)	16/92 (17.4%)
Physical activity	18/44 (40.9%)	14/26 (53.8%)	43/85 (50.6%)	41/92 (44.6%)
Association life	21/44 (47.7%)	11/26 (42.3%)	28/85 (32.9%)	17/92 (18.5%)
Hobbies	6/44 (13.6%)	4/26 (15.4%)	13/85 (15.3%)	12/92 (13.0%)
Art, literature, and music	2/44 (4.5%)	8/26 (30.8%)	24/85 (28.2%)	19/92 (20.7%)
Religious activity	4/44 (9.1%)	1/26 (3.8%)	6/85 (7.1%)	13/92 (14.1%)
Being in nature	25/44 (56.8%)	12/26 (46.2%)	49/85 (57.6%)	53/92 (57.6%)
Online support group	1/44 (2.3%)	5/26 (19.2%)	13/85 (15.3%)	35/92 (38.0%)
Meeting friends	21/44 (47.7%)	14/26 (53.8%)	53/85 (62.4%)	50/92 (54.3%)
Everyday conversations	20/44 (45.5%)	15/26 (57.7%)	43/85 (50.6%)	51/92 (55.4%)
Social media	1/44 (2.3%)	3/26 (11.5%)	13/85 (15.3%)	15/92 (16.3%)
Other	2/44 (4.5%)	2/26 (7.7%)	9/85 (10.6%)	13/92 (14.1%)

## Discussion

### Discussion of the results

The aim of the present study was to investigate support and coping with grief following the loss of a significant other in the case of ED and UED in the context of contemporary Sweden. Certain aspects emerged that highlight how support in grief is handled from a social and religious point of view.

The social network, including family, relatives, and friends, was found to be the most common source of support for those experiencing grief, in line with previous findings. The effectiveness of this support can depend on function, capacity, relationships within the family and network, and the gap between the bereaved person’s needs and the support offered, all in line with earlier findings.^29,30,40^ The most requested form of support was emotional and emotional support combined with practical support. Emotional support is mainly presented in the form of conversations, on an individual basis, or within a bereavement group. A greater need for all forms of support, both emotional and practical, including advice and information, was expressed by those who had experienced a UED than those who had experienced an ED.^41,42^ This difference may be explained by the fact that those who have experienced an ED would have had more time to prepare themselves.^43^ There was a gender difference as men did not perceive the need for support to the same extent as women did. There was significant agreement among participants that support should be offered proactively at the time of death, with many stating that they had not felt capable of seeking contacts outside their network. Most of the participants in this study had experienced a loss more than a year ago and were now able to retrospectively identify the support they needed. In particular, some expressed the expectation that the healthcare system should provide information about the cause of the death as well as offer emotional support to those experiencing grief.^40^ It can be a challenge for healthcare professionals to determine appropriate measures to support those experiencing grief in the wake of a death. Follow-up meetings have previously been suggested as an effective method.^44,45^

In this study, most of the participants stated that they felt the support they did receive was helpful. Although the need for support can vary during bereavement, so can the form of support needed and its source. Those who experienced a UED received more support from people outside of their family and friends, such as co-workers and support groups than those who experienced an ED. A potential reason for this can be the age of the bereaved, their employment status, and the nature of their social network. A UED is often experienced as traumatic, which can result in certain expectations and needs for different forms of support. In this study, most participants felt they had received the support they needed. However, some also reported a lack of support and information needed in dealing with their loss.^45,46,47^ There is always a risk that the bereaved person feels some lack of support, in some way, and it is important that they are allowed to express the support they need.^48–50^ Women felt more supported than men in various forms of support groups.

This study describes how grief influences parts of the social and health life situation in different ways. Most of the participants described there being little impact on their financial and housing situation after the death; though there was a slightly greater impact on women than on men. However, this can vary greatly, depending on how the person’s finances are structured and whether they were dependent on the income of the deceased.^51^ Grief can influence health outcomes from both a physical and a psychological perspective. There was a significant difference in the impacts on health between participants who experienced a UED compared to an ED. Participants who had experienced a UED were more likely to report that their overall health had become worse or much worse than participants who had experienced an ED. This can be explained by the varied individual experiences of a UED. Even if a death is expected, the timing may be unexpected. How an individual handles a situation such as a UED can depend on how they feel at the time, both physically and psychologically, as well as their resilience in coping with difficult life events and the perceived level of support available to them. However, it should be taken into consideration that a UED always poses a risk of a change in health and a difficult grieving period.^52,53^

Death and the subsequent grieving process can affect the bereaved person’s living conditions and change their relationship with others. In this study, more women than men described the need for practical support in their living situation. Depending on the connection to the deceased, the social relationships of the bereaved person can change significantly, depending on how others handle the death and its consequences. Some consequences can also contribute to social changes, both practical and emotional, how they are experienced and the bereaved person’s capacity to cope with and manage the new situation. This will also depend on how the bereaved person perceives the support they receive from others. The less support they perceive, the more isolated they may feel in the grieving process.

Support to deal with grief can depend on both social and religious perspectives of death and grief. The coping styles were described earlier as a dual process shifting between facing the loss and focusing beyond the loss, both individually and in a family context.^24,25^ In this study, participants described being able to discuss and talk about their grief, either individually or in a group, and activities as being helpful in dealing with grief. Partaking in activities was seen as healing in itself, but being in an environment that provided detachment from thoughts of death and grief, for example at work or in an association, was also important. A number of participants highlighted pets as a source of comfort in grief, as well as assistance in coping with difficult situations. Additionally, being in nature was appreciated as a form of support. Table 3, “What has helped you in grief?” can provide suggestions for professionals as well as significant others on what may be helpful for a grieving person. A study on coping mechanisms used by bereaved parents in Sweden revealed that talking to others, pondering on the meaning of life alone, and connecting with nature are frequently used secular existential coping methods.^54^ The religious historian David Thurfjell recently argued that nature has not only become a site of sanctity and contemplation for the Swedish population but also holds significance in replacing Christianity’s transcendent God with immanent sacredness. In line with Thurfjell’s argument, it is not particularly surprising that walks in the forest and spending time in nature stand out in the survey responses as specific sources of tranquility and contemplation when coping with grief.^55^

The study also included variables measuring religious belonging, belief in God/a higher power, religious and spiritual practice, and religious attendance as sources of religious coping.^26^ The relatively high occurrence of spiritual belief is not surprising in relation to the Swedish population in general.^56^ The high level of self-directing coping styles reported in times of crisis, in which the respondent trusts their own ability to handle difficulties in life, does not rule out religious belonging or a spiritual outlook on life. Importantly, the self-directing coping style does not exclude social needs or support from others, although it might be seen as an indicator of a high level of individualism and secular/rational values, in contrast to more traditional values in which religion and belief in God/a higher power usually occupies a more central place in people’s lives. Belonging to a religion does not automatically mean that a person puts their trust in God/a higher power in times of distress and, conversely, many of those who reported using a self-directing coping style belonged to a religion. This implies that neither religious/spiritual belief nor affiliation precludes a self-directing coping style. In essence, there is no inherent contradiction between faith in God, religious affiliation, and a self-directing coping style. However, only a small minority stated it as an important factor compared to the majority who stated that they identified meeting friends, everyday conversations, and spending time in nature as crucial support in grief. Furthermore, religious belonging does not necessarily indicate a belief in God, which is also reflected in the number of participants who reported being members of the Church of Sweden compared to a belief in God.^56^ Additionally, it is important to actively challenge and sometimes dissolve the boundaries between religious, spiritual, and other practices when looking to understand activities that provide meaning during grief in the wake of a death. This aligns with current research in religious studies, in which, for instance, nature has been investigated as a central aspect of the “religion” of Swedes, as reflected in this study.^55^ Furthermore, other significant beliefs and practices that are typically not labeled as either religious or spiritual should be included to achieve a more holistic understanding of what aids individuals in grief following the loss of a significant other.

### Strength and limitations

This study has both limitations and strengths. One limitation is that it is not known how many people received the survey. Those who responded are likely to have been keen to discuss their grief; for this reason, their perspectives may not be entirely representative. Regarding the religious composition of the study, it is evident that the sample does not adequately reflect the overall religious demographics in Sweden as participants from minority religious groups are almost absent. There could be several reasons for the absence of these groups from the survey, including linguistic barriers, as well as the fact that they may not have been reached through the distribution channels of the study. A strength of the study is that the participants had all experienced the death more than six months earlier, which means that they had had the opportunity to reflect on how the grief affected them and their life situation.

## Conclusion

The study results can contribute to a greater understanding of bereavement following the loss of a significant other, and it reveals the need for support, coping strategies, and faith following the loss of a significant other. The study also shows the impacts of an ED or a UED on the support and life circumstances of the bereaved. Family, relatives, and friends are the most common sources of support for those experiencing grief. The most requested forms of support were emotional and emotional combined with practical support. The value of different forms of support received, social and religious, can differ between bereaved persons depending on whether they experienced the support as helpful.

The study also identifies that support and coping with grief may differ between women and men. Grief following the loss of a significant other can depend on whether the death is experienced as an ED or a UED. It can also influence the life circumstances of the bereaved person in many ways and how the bereaved person interacts with others. Pets and nature were both highlighted as sources of support and comfort when dealing with difficult life circumstances. Self-directing coping was the most common strategy for handling difficulties in life, although this often incorporated religious belonging or a spiritual outlook on life. This self-directing coping style does not exclude social needs or the support of others. Based on current grief theories that emphasize grief as an individual process for the vast majority, these findings can contribute to the understanding of bereaved persons. Further research is needed on how circumstances surrounding death affect feelings and experiences, as well as how different beliefs and practices are expressed and help those experiencing grief after the loss of a significant other.

## Supplemental Material

sj-docx-1-pcr-10.1177_26323524241275699 – Supplemental material for Understanding the needs for support and coping strategies in grief following the loss of a significant other: insights from a cross-sectional survey in Sweden Supplemental material, sj-docx-1-pcr-10.1177_26323524241275699 for Understanding the needs for support and coping strategies in grief following the loss of a significant other: insights from a cross-sectional survey in Sweden by Inger Benkel, Johanna Skoglund, Daniel Enstedt, Ylva Hård af Segerstad, Joakim Öhlén and Stina Nyblom in Palliative Care and Social Practice

## Declarations

**Ethics approval and consent to participate:** Consent to participate was given when the participants returned the survey. Participation in the study was anonymous and could be ended at any time. Ethical approval to conduct the study was obtained from the Swedish Ethical Review Authority (2021-02992, 2021-06-29).

**Consent for publication:** The information letter described that the study would be published and that through their submission, the participants approved publication.

**Availability of data and materials:** Data are available on request.
